# Disparities in Survival Outcomes of Out-of-Hospital Cardiac Arrest Patients between Urban and Rural Areas and the Identification of Modifiable Factors in an Area of South Korea

**DOI:** 10.3390/jcm11144248

**Published:** 2022-07-21

**Authors:** Song Yi Park, Daesung Lim, Seong Chun Kim, Ji Ho Ryu, Yong Hwan Kim, Byungho Choi, Sun Hyu Kim

**Affiliations:** 1 Department of Emergency Medicine, Dong-A University College of Medicine, Dong-A University Hospital, Busan 48114, Korea; capesong@naver.com; 2 Department of Emergency Medicine, Seoul Medical Center, Seoul 03080, Korea; daesung2@gmail.com; 3 Department of Emergency Medicine, Kang-II Hospital, Gimhae 70712, Korea; gsimem@naver.com; 4 Department of Emergency Medicine, Pusan National University College of Medicine, Pusan National University Yangsan Hospital, Busan 50612, Korea; pnuyhem@gmail.com; 5 Department of Emergency Medicine, Samsung Changwon Hospital, Sungkyunkwan University School of Medicine, Changwon 51353, Korea; suka1212@naver.com; 6 Department of Emergency Medicine, University of Ulsan College of Medicine, Ulsan University Hospital, Ulsan 44033, Korea; byungho2000@gmail.com

**Keywords:** emergency medical services, out-of-hospital cardiac arrest, urban populations, rural health services

## Abstract

This retrospective study aimed to compare the survival outcomes of adult out-of-hospital cardiac arrest (OHCA) patients between urban (Busan, Ulsan, Changwon) and rural (Gyeongnam) areas in South Korea and identify modifiable factors in the chain of survival. The primary and secondary outcomes were survival to discharge and modifiable factors in the chain of survival were identified using logistic regression analysis. In total, 1954 patients were analyzed. The survival to discharge rates in the whole region and in urban and rural areas were 6.9%, 8.7% (Busan 8.7%, Ulsan 10.3%, Changwon 7.2%), and 3.4%, respectively. In the urban group, modifiable factors associated with survival to discharge were no advanced airway management (adjusted odds ratio (aOR) 2.065, 95% confidence interval (CI): 1.138–3.747), no mechanical chest compression (aOR 3.932, 95% CI: 2.015–7.674), and an emergency medical service (EMS) transport time of more than 8 min (aOR 3.521, 95% CI: 2.075–5.975). In the rural group, modifiable factors included an EMS scene time of more than 15 min (aOR 0.076, 95% CI: 0.006–0.883) and an EMS transport time of more than 8 min (aOR 4.741, 95% CI: 1.035–21.706). To improve survival outcomes, dedicated resources and attention to EMS practices and transport time in urban areas and EMS scene and transport times in rural areas are needed.

## 1. Introduction

Out-of-hospital cardiac arrest (OHCA) is a major public health concern. Survival outcomes of OHCA patients vary by country. The proportion of surviving patients discharged after OHCA in South Korea in 2015 was 9.6% [1]. Many studies have reported factors associated with survival following OHCA, such as patient age, comorbidity, initial cardiac rhythm, witness status, bystander cardiopulmonary resuscitation (CPR), bystander defibrillation, emergency medical service (EMS)-provided advanced life support, EMS processing time, and in-hospital interventions [2,3,4]. Among these factors, patient age, initial cardiac rhythm, and witness status are nonmodifiable; the others are modifiable and are components of the chain of survival.

Factors related to the chain of survival vary depending on the level of urbanization, even within the same country [5,6]. In a Saudi Arabian study, the median response time of ambulances in rural areas was 1.3 times that in the urban area [7]. A study from Japan reported that acute myocardial infarction patients in rural areas were less likely to be directly transported to intervention-capable facilities, resulting in a longer time to primary intervention than that in metropolitan areas [8].

Differences in the chain of survival-related factors between urban and rural areas seem to lead to disparities in the survival outcomes of OHCA patients. Urban patients who experienced bystander-witnessed cardiac arrest were three times more likely to arrive at an emergency department (ED) with cardiac output [9]. OHCA patients in the urban group had an approximately 1.5-fold higher probability of survival to discharge than those in the rural group, with a significant difference [10].

If there is a difference in urbanization levels within the region served by one EMS system, the survival outcomes of OHCA patients and the modifiable factors in the chain of survival will differ between the urban and rural areas. Identifying weak links in the regional chain of survival is essential for improving the regional EMS system and OHCA patient survival outcomes. However, few studies have compared urban and rural survival outcomes and identified weak links in the chain of survival to narrow this gap. Therefore, this study aimed to compare the survival outcomes of OHCA patients and identify modifiable factors in the chain of survival between urban and rural areas in a region of South Korea.

## 2. Material and Methods

### 2.1. Study Design and Ethical Approval

This retrospective observational study was conducted in the Busan, Ulsan, Changwon, and Gyeongnam regions in South Korea. We compared the survival outcomes and identified modifiable factors associated with survival after OHCA in residents of urban and rural areas of this region using prehospital EMS data, which were collected from 1 November 2019 to 31 January 2020 and from 1 November 2020 to 31 January 2021. The study was approved by the Institutional Review Board of Dong-A University Hospital (approval no. DAUHIRB-EXP-22-036). The requirement for informed patient consent was waived because the study was a retrospective analysis of existing data that did not contain personal information at the time the data were provided.

### 2.2. Study Setting

The Busan, Ulsan, Changwon, and Gyeongnam regions are located along the southeastern coast of Korea. Historically, these areas have been considered to have similar cultures and actively interact with each other. This region consists of two metropolitan cities (Busan and Ulsan, Korea), one city (Changwon, Korea), and one province (Gyeongnam, Korea), with a total population of 7.92 million, spread over almost 12,369 km^2^. The population densities (persons/km^2^) were 4349 in Busan, 1069 in Ulsan, 1376 in Changwon, and 316.2 in Gyeongnam in 2020 [11]. We defined Busan, Ulsan, and Changwon as urban areas and Gyeongnam as a rural area, referring to the level of urbanization and population density.

The EMS system in the region is government-based and single-tiered. The service provides basic-to-intermediate levels of EMS care, such as supraglottic airway device insertion, tracheal intubation, and basic life support (BLS). The EMS system is based out of fire agency headquarters in each region. The EMS resuscitation protocol includes multiple dispatches, on-site CPR, and transportation of patients to the nearest ED in an ambulance while continuing CPR. EMS providers cannot stop CPR until the return of spontaneous circulation (ROSC) or without medical oversight by medical directors, either on site or during transportation to the ED. Only physicians in hospital EDs can declare death. Ambulances staffed with a physician are not available. Most EMS teams in urban areas consist of three EMS providers, including at least one emergency medical technician. Among the practices of EMS providers, advanced airway management, intravenous cannulation, fluid and drug administration, and withholding/withdrawal of resuscitation are performed under medical oversight by medical directors. There is only one medical oversight association for the entire region, and all medical directors are local emergency physicians [12].

### 2.3. Study Population

The inclusion criteria were as follows: patients with OHCA and resuscitation attempted by EMS providers during the study period. The exclusion criteria were as follows: age less than 18 years old or arrest due to trauma, intoxication, or drowning. Patients were excluded if resuscitation was not attempted due to obvious signs of death or if they had a valid do not resuscitate (DNR) order. Patients were also excluded if arrest occurred in a health care facility staffed with a physician.

### 2.4. Data Sources and Collection

Regional fire agencies collect and manage all prehospital EMS dispatch data electronically. The EMS providers file a prehospital cardiac arrest patient care report for resuscitation cases. We collected anonymous prehospital data from the four regional fire agency headquarters by submitting a research proposal. We collected in-hospital data from 76 treating-hospital EDs by directly contacting the institutions.

The collected variables included patient, bystander, and EMS factors and survival outcomes. Patient variables included age, sex, and medical history (hypertension, diabetes, stroke, cardiac disease, pulmonary disease, liver disease, renal disease, or malignancy). Bystander variables included arrest location (residence, public, nursing facility, or ambulance), EMS call time (08:00–16:00, 16:00–24:00, or 00:00–08:00), witness status (bystander-witnessed), bystander CPR, and bystander defibrillation. EMS variables included initial cardiac rhythm (shockable and nonshockable), advanced airway management type (I-gel/supraglottic airway, tracheal intubation, or no advanced airway management), the use of a mechanical chest compression device, adrenaline (epinephrine) administration, and EMS processing time (response, scene, and transport times). EMS response, scene, and transport times were defined as the times from the call to EMS to EMS arrival at the scene, from EMS arrival at the scene to EMS departure from the scene, and from EMS departure from the scene to EMS arrival at the ED, respectively. Survival outcomes included ROSC at any time, survival at admission, survival to discharge, and favorable neurological outcomes.

### 2.5. Outcome Measures

The primary outcome was survival to discharge after OHCA in the urban and rural groups. The secondary outcome was modifiable factors associated with survival after OHCA in the urban and rural groups.

### 2.6. Statistical Analysis

The data are presented as frequencies with percentages for categorical variables and means ± standard deviations (SDs) and medians (25th–75th percentiles) for continuous variables. Differences in study population characteristics were compared among subgroups with the Chi-squared test or Fisher’s exact test for categorical variables. An independent *t*-test or the Mann–Whitney U test was also employed as appropriate. The Shapiro–Wilk test was used to evaluate distribution normality. Univariate and multivariate analyses using logistic regression models were performed to identify factors independently associated with the outcome variable. The cutoffs for the response, scene, and transport times were reported in previous studies [13,14,15,16]. Crude odds ratios (ORs) and adjusted odds ratios (aORs) were estimated to investigate the association between the region (urban or rural) and the outcome variable of interest. Variables showing a univariate association with the outcome (at *p* < 0.10) were included in stepwise backward multivariate logistic models to adjust for confounders. Associations between an outcome variable and region based on the univariate and multivariate conditional logistic regression analyses were identified with ORs and 95% confidence intervals (CIs) after controlling for confounding factors. Propensity matching was performed to reduce the influences of confounding variables on survival outcomes. Confounding variables included in the propensity model were age, sex, medical history, arrest location, EMS call time, witness status, and initial cardiac rhythm at the scene. The selection criteria for matching variables were nonmodifiable factors associated with the OHCA survival rate. Matching was performed using a greedy algorithm with the estimated propensity score. An 8 to 1 digit greedy matching algorithm was used to match an individual urban patient to each rural patient according to the propensity score using SAS PROC LOGISTIC, and a greedy matching algorithm was used to match cases to controls (SAS macro code: http://www2.sas.com/proceedings/sugi29/165-29.pdf, accessed on 25 April 2020) [17]. Sensitivity analyses (Busan, Ulsan, and Changwon vs. Gyeongnam) were performed to assess the robustness of the findings or conclusions based on the primary data analyses. Formal sample size calculation was not performed due to the nature of the study design. All statistical analyses were carried out using SPSS 26.0 (IBM Corp., Armonk, NY, USA, released 2019. IBM SPSS Statistics for Windows, Version 26.0. Armonk, NY: IBM Corp) and SAS 9.3 (SAS Institute, Cary, NC, USA). *p* values less than 0.05 were considered indicative of statistical significance.

## 3. Results

### 3.1. Characteristics of the Study Population

**Figure 1:**
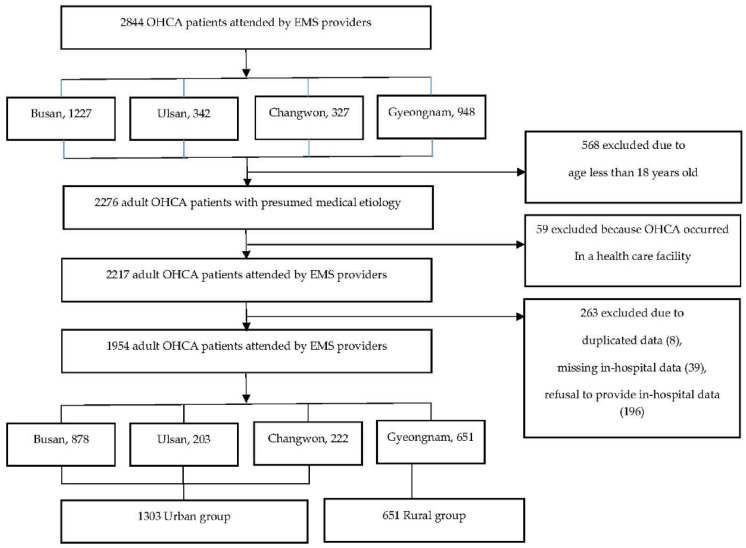
Flowchart of the study population.

A total of 2844 eligible adult OHCA patients were identified during the study period. Six hundred twenty-seven patients who did not fulfill the inclusion criteria were excluded, and 263 patients with duplicated data, missing data, apparent death, or a DNR order or who refused to allow the use of in-hospital data were excluded. Ultimately, 1954 patients were included in the analysis (Figure 1). The mean age was 70.7 years, and 62.6% of participants were male. Approximately 16.0% of the patients had cardiac disease. Most OHCAs occurred at the patient’s residence (69.2%). The initial rhythm was mostly nonshockable (89.9%). Fewer than two-thirds (59.3%) of the patients received bystander CPR, and a few patients (5.2%) received defibrillation. The median (interquartile range) EMS response, scene, and transport times were 7.0 (6.0–10.0), 14.0 (10.0–18.0), and 6.0 (4.0–11.0) minutes, respectively. The median EMS response (7.0 vs. 9.0) and transport (6.0 vs. 8.0) times were shorter in the urban areas than in the rural area. However, the median EMS scene time between the urban and rural areas was not different (Table 1).

### 3.2. Survival to Discharge after OHCA

The survival to discharge rate in the urban group was 8.7%, which was significantly higher than 3.4% in the rural group (*p* value < 0.001) (Table 2).

### 3.3. Factors Associated with Survival to Discharge after OHCA

In the total study population, the modifiable factors independently associated with survival to discharge included no advanced airway management (aOR 1.877, 95% CI: 1.067–3.301), no mechanical chest compression (aOR 3.772, 95% CI: 2.022–7.037), and an EMS transport time of more than 8 min (aOR 3.202, 95% CI: 1.981–5.175) (Table 3).

In the urban group, the modifiable factors associated with survival to discharge were no advanced airway management (aOR 2.065, 95% CI: 1.138–3.747), no mechanical chest compression (aOR 3.932, 95% CI: 2.015–7.674), and an EMS transport time of more than 8 min (aOR 3.521, 95% CI: 2.075–5.975) (Table 4).

In the rural group, the modifiable factors associated with survival to discharge included an EMS scene time of more than 15 min (aOR 0.076, 95% CI: 0.006–0.883) and an EMS transport time of more than 8 min (aOR 4.741, 95% CI: 1.035–21.706) (Table 5).

## 4. Discussion

This study aimed to compare the survival outcomes of OHCA patients between urban and rural areas and identify modifiable factors in the chain of survival. In this region, the survival to discharge rate in the urban group was more than twice that in the rural group. EMS transport time was a modifiable factor in both groups. However, EMS practices (advanced airway management and mechanical chest compression) were the only associated factors in the urban group, and EMS scene time was the only associated factor in the rural group.

In this study, the urban group had more factors that were associated with favorable survival outcomes (age, public arrest, bystander CPR, bystander defibrillation, and EMS response time but not initial cardiac rhythm). Previous studies have found that advanced age and delayed EMS response time were strongly associated with poor OHCA outcomes [18,19]. In contrast, bystander CPR and bystander defibrillation were associated with increased survival after OHCA [20,21]. It is well established that a shockable rhythm is a strong predictor of favorable survival outcomes after OHCA [21]. The higher rate of survival in the urban group in this study is speculated to be due to the integral contributions of these differences.

In this region, bystander CPR and defibrillation were not associated with survival outcomes following OHCA. Rather, EMS practices (advanced airway management and mechanical chest compression) and EMS transport time were survival-associated factors. However, it cannot be assumed that bystander CPR and defibrillation are less important than EMS-related factors because this result may be due to the relatively small sample size. The proportion of bystander defibrillation was 6.95% in the urban group and 1.7% in the rural group. This may have resulted to a low survival rate in the rural group even though the proportion of patients with a shockable rhythm was higher than that in the urban group (8.9% vs. 12.6%). These findings suggest that public education about bystander CPR and defibrillation is essential. Nevertheless, more attention and resources need to be allocated to the EMS system in the region to improve the survival rate of OHCA patients.

We conducted propensity score-matched analysis, in which patients in the urban and rural groups were matched according to unmodifiable factors affecting OHCA survival; such factors included age, sex, medical history, arrest location, EMS call time, witness status, and initial cardiac rhythm (Appendix A, 1,2). The survival gap increased to 6.7% (urban 10.1% vs. rural 3.4%, *p* < 0.001). This suggests that modifiable factors affect survival outcomes more in urban areas than in rural areas. This reaffirms the importance of identifying modifiable factors in the chain of survival for urban patients. However, for rural patients, EMS scene and transport times were the only associated factors. This may imply that other factors that were not measured in detail in this study, such as in-hospital intervention factors, may significantly impact survival outcomes.

Outcomes associated with advanced airway management and bag-mask ventilation intervention are highly dependent on the skillset and experience of the EMS providers. Thus, recommendations for the application of advanced airway strategies are not defined in global guidelines [22]. Studies have shown that survival rates were higher among patients who received no advanced airway management than among patients who received endotracheal intubation or supraglottic airway device insertion [23,24,25,26]. Our finding was consistent with the results of previous studies. However, we also consider that this finding reflects unmeasured and immeasurable confounders [26]. The ‘no advanced airway group’ may have included patients who regained spontaneous respiration or consciousness during EMS treatment or who suffered from failed or mispositioned airway device placement, or airway management practices may have prevented EMS providers from delivering high-quality BLS.

A recent systematic review of studies on mechanical chest compression found no evidence of improved survival with good neurological outcomes in OHCA patients [27]. However, there are many residences on hills in this region. Hence, EMS providers sometimes have to transport OHCA patients over stairs. In these cases, a mechanical chest compression device is the only alternative. Additionally, EMS providers may have used mechanical chest compression devices due to concerns about coronavirus disease 2019 (COVID-19) during the study period. In this situation, mechanical chest compression was not a modifiable factor. However, the OR for survival in the no mechanical chest compression group was high. Further study is needed to confirm the quality of mechanical chest compression and its impact in this region.

In this study, an EMS transport time of more than 8 min was positively associated with survival outcomes. Recent studies showed that EMS transport time was not associated with survival to discharge or neurological outcomes at hospital discharge in adult OHCA patients [28,29]. On the other hand, a study found that a longer transport time interval adversely affected the likelihood of good neurological recovery among OHCA patients [30]. We retrospectively verified the data and found that some of the patients with ROSC before ED arrival were transported to a higher-level ED that was more capable of providing postcardiac arrest care than the closest ED; thus, their EMS transport times were unusually long. There is no clear regional guideline suggesting the ED to which the EMS provider should transport these patients. Considering the risk of rearrest in such patients, the regional guidelines should be re-evaluated and revised.

This study has several limitations. First, the findings of this study cannot be generalized to other regions with different EMS systems. However, we believe that this study has value in this region. Second, the urban group was composed of patients from three cities (Busan, Ulsan, and Changwon), and patients from Busan made up 45% of the total study population. However, we observed consistent results in the sensitivity analyses between Busan and the rural region, Ulsan and the rural region, and Changwon and the rural region (Appendix A, 3–5). Third, in-hospital interventions, such as therapeutic hypothermia or coronary angiography, were not considered. In rural areas, in-hospital interventions are expected to affect survival outcomes. Fourth, this study population included patients who experienced OHCA during the COVID-19 pandemic. The prehospital EMS and OHCA survival outcomes during this period will be different from those before the pandemic. However, we have not adjusted for these changes.

## 5. Conclusions

In the Busan, Ulsan, Changwon, and Gyeongnam areas in South Korea, there is a more than two-fold difference in OHCA survival outcomes between urban and rural residents. To improve the survival outcomes of OHCA patients in the region, it is necessary to monitor and allocate resources to improve EMS practices and transport time in urban areas and EMS scene and transport times in rural areas. In addition, studies should be conducted to monitor whether survival outcomes in OHCA patients improve when these factors in the chain of survival improve.

## Figures and Tables

**Table 1:** Characteristics of the study population.

Variables	Total (*n* = 1954)		Urban (*n* = 1303)		Urban (*n* = 1303)	Rural (*n* = 651)	*p* Value (Urban vs. Rural)
		Busan (*n* = 878)	Ulsan (*n* = 203)	Changwon (*n* = 222)		Gyeongnam (*n* = 651)	
Patient variables							
Age, mean ±SD	70.7 ± 15.0	70.2 ± 15.0	68.8 ± 16.3	71.6 ± 14.4	70.2 ± 15.1	71.8 ± 14.9	0.030 ^1^
Age median (Q1–Q3)	74.0 (61.0–82.0)	73.0 (61.0–81.0)	70.0 (56.0–82.0)	76.0 (64.0–81.0)	73.0 (61.0–81.0)	76.0 (61.0–83.0)	
≤65 years	633 (32.4)	288 (32.8)	84 (41.4)	60 (27.0)	432 (33.2)	201 (30.9)	0.310 ^2^
>65 years	1321 (67.6)	590 (67.2)	119 (58.6)	162 (73.0)	871 (66.8)	450 (69.1)	
Sex (male %)	1224 (62.6)	579 (65.9)	120 (59.1)	147 (66.2)	846 (64.9)	378 (58.1)	0.003 ^2^
Medical history							
hypertension	536 (27.7)	233 (26.5)	50 (27.2)	73 (32.9)	356 (27.7)	180 (27.6)	0.972 ^2^
diabetes	363 (18.8)	150 (17.1)	37 (20.1)	53 (23.9)	240 (18.7)	123 (18.9)	0.914 ^2^
stroke	151 (7.8)	64 (7.3)	16 (8.7)	18 (8.1)	98 (7.6)	53 (8.1)	0.693 ^2^
cardiac disease	310 (16.0)	124 (14.1)	37 (20.1)	40 (18.0)	201 (15.7)	109 (16.7)	0.537 ^2^
pulmonary disease	141 (7.3)	72 (8.2)	11 (6.0)	14 (6.3)	97 (7.6)	44 (6.8)	0.525 ^2^
liver disease	31 (1.6)	13 (1.5)	3 (1.6)	4 (1.8)	20 (1.6)	11 (1.7)	0.827 ^2^
renal disease	78 (4.0)	34 (3.9)	8 (4.3)	9 (4.1)	51 (4.0)	27 (4.1)	0.853 ^2^
malignancy	197 (10.2)	81 (9.2)	22 (12.0)	31 (14.0)	134 (10.4)	63 (9.7)	0.602 ^2^
Bystander variables							
Arrest location							
residence (home)	1353 (69.2)	596 (67.9)	144 (70.9)	163 (73.4)	903 (69.3)	450 (69.1)	0.011 ^2^
public	396 (20.3)	196 (22.3)	39 (19.2)	40 (18.0)	275 (21.1)	121 (18.6)	
nursing facility	78 (4.0)	28 (3.2)	5 (2.5)	6 (2.7)	39 (3.0)	39 (6.0)	
ambulance	127 (6.5)	58 (6.6)	15 (7.4)	13 (5.9)	86 (6.6)	41 (6.3)	
EMS call time (24 h)							
08:00–16:00	747 (38.2)	338 (38.5)	75 (36.9)	86 (38.7)	499 (38.3)	248 (38.1)	0.453 ^2^
16:00–24:00	725 (37.1)	342 (39.0)	71 (35.0)	80 (36.0)	493 (37.8)	232 (35.6)	
00:00–08:00	482 (24.7)	198 (22.6)	57 (28.1)	56 (25.2)	311 (23.9)	171 (26.3)	
Bystander-witnessed	819 (44.9)	353 (43.2)	93 (48.7)	90 (41.7)	536 (43.8)	283 (47.2)	0.173 ^2^
Bystander CPR	1069 (59.3)	516 (61.7)	117 (62.9)	112 (55.7)	745 (60.9)	324 (56.0)	0.045 ^2^
Bystander defibrillation	96 (5.2)	66 (7.7)	15 (7.9)	5 (2.5)	86 (6.9)	10 (1.7)	<0.001 ^2^
EMS variables							
Initial cardiac rhythm							
shockable	197 (10.1)	60 (6.9)	33 (16.4)	23 (10.4)	116 (8.9)	81 (12.6)	0.013 ^2^
nonshockable	1745 (89.9)	815 (93.1)	168 (83.6)	198 (89.6)	1181 (91.1)	564 (87.4)	
Advanced airway							
I-gel/supraglottic	1413 (81.4)	684 (85.1)	44 (74.6)	121 (54.5)	849 (78.2)	564 (86.6)	<0.001 ^2^
tracheal intubation	53 (3.1)	32 (4.0)	2 (3.4)	3 (1.4)	37 (3.4)	16 (2.5)	
no advanced airway	270 (15.6)	88 (10.9)	13 (22.0)	98 (44.1)	199 (18.3)	71 (10.9)	
Mechanical chest compression	745 (38.2)	266 (30.3)	113 (55.7)	137 (62.0)	516 (39.7)	229 (35.2)	0.054 ^2^
Epinephrine administration	337 (17.3)	98 (11.2)	34 (16.7)	82 (36.9)	214 (16.4)	123 (19.0)	0.150 ^2^
EMS process time (min)							
EMS response time,							
mean ±SD	9.0 ± 6.1	8.3 ± 5.4	7.8 ± 3.2	8.8 ± 6.5	8.3 ± 5.3	10.4 ± 7.3	<0.001 ^1^
median (Q1–Q3)	7.0 (6.0–10.0)	7.0 (6.0–9.0)	7.0 (6.0–9.3)	7.0 (5.0–10.0)	7.0 (5.0–9.0)	9.0 (6.0–13.0)	
≤8 min	1211 (62.0)	600 (68.3)	130 (64.4)	156 (70.3)	886 (68.0)	325 (49.9)	<0.001 ^2^
>8 min	742 (38.0)	278 (31.7)	72 (35.6)	66 (29.7)	416 (32.0)	326 (50.1)	
EMS scene time,							
mean ±SD	14.4 ± 6.4	13.9 ± 5.9	13.8 ± 5.3	17.3 ± 6.9	14.5 ± 6.1	14.1 ± 7.0	0.086 ^1^
median (Q1–Q3)	14.0 (10.0–18.0)	13.0 (10.0–17.0)	13.0 (10.0–17.0)	17.0 (13.0–21.0)	14.0 (11.0–18.0)	14.0 (9.0–18.0)	
≤15 min	1185 (61.1)	563 (65.2)	128 (63.1)	88 (39.6)	779 (60.4)	406 (62.6)	0.365 ^2^
>15 min	753 (38.9)	301 (34.8)	75 (36.9)	134 (60.4)	510 (39.6)	243 (37.4)	
EMS transport time,							
mean ±SD	9.4 ± 11.0	9.5 ± 12.6	5.8 ± 4.2	7.7 ± 7.2	8.6 ± 11.0	11.1 ± 11.0	<0.001 ^1^
median (Q1–Q3)	6.0 (4.0–11.0)	6.0 (4.0–10.0)	5.0 (3.0–7.0)	6.0 (4.0–10.0)	6.0 (4.0–9.0)	8.0 (4.0–15.0)	
≤8 min	1268 (64.9)	601 (68.5)	164 (80.8)	153 (68.9)	918 (70.5)	350 (53.8)	<0.001 ^2^
>8 min	686 (35.1)	277 (31.5)	39 (19.2)	69 (31.1)	385 (29.5)	301 (46.2)	

Variables are presented as the mean ± standard deviation, median (25th–75th percentile), or number (%). ^1^ *p* value was derived from the Mann–Whitney U test. ^2^ *p* value was derived from the Chi-squared test. Shapiro–Wilk’s test was employed to test the normality assumption.

**Table 2:** Survival outcomes of the study population.

Variables	Total (*n* = 1954)	Urban (*n* = 1303)			Urban (*n* = 1303)	Rural (*n* = 651)	*p* Value (Urban vs. Rural)
		Busan (*n* = 878)	Ulsan (*n* = 203)	Changwon (*n* = 222)		Gyeongnam (*n* = 651)	
ROSC at any time	562 (28.8)	284 (32.3)	51 (25.1)	65 (29.3)	400 (30.7)	162 (24.9)	<0.001 ^2^
Survival at admission	397 (20.3)	201 (22.9)	43 (21.2)	42 (18.9)	286 (21.9)	111 (17.1)	0.011 ^1^
Survival to discharge	135 (6.9)	76 (8.7)	21 (10.3)	16 (7.2)	113 (8.7)	22 (3.4)	<0.001 ^1^
Favorable neurological outcome	89 (4.6)	42 (4.8)	15 (7.4)	13 (5.9)	70 (5.4)	19 (2.9)	0.019 ^1^

Values are presented as the number of patients surviving to discharge with the percentage in parentheses. ^1^ *p* value was derived from the Chi-squared test. ^2^ *p* value was derived from Fisher’s exact test.

**Table 3:** Factors associated with survival to discharge after OHCA in the total study population.

Variable	Univariate Analysis			Multivariate Analysis		
	OR	95% CI	*p* Value	OR	95% CI	*p* Value
Urban	2.715	(1.702, 4.330)	<0.001	4.471	(2.389, 8.368)	<0.001
Rural	Ref.	-		Ref.	-	
Patient variables						
Age ≤65 years	Ref.	-	<0.001	Ref.	-	<0.001
Age >65 years	0.259	(0.169, 0.395)		0.291	(0.176, 0.480)	
Sex (male)	2.094	(1.387, 3.161)	<0.001	1.297	(0.747, 2.252)	0.355
Sex (female)	Ref.	-		Ref.	-	
Medical history						
cardiac disease (+)	Ref.	-	0.024	Ref.	-	0.045
cardiac disease (−)	0.615	(0.404, 0.937)		0.553	(0.310, 0.987)	
Bystander variables						
Arrest location						
residence (home)	0.260	(0.178, 0.379)	<0.001	0.508	(0.304, 0.849)	0.010
Public	Ref.	-		Ref.	-	
Others	0.442	(0.244, 0.800)	0.007	0.179	(0.047, 0.687)	0.012
EMS call time (24 h)						
08:00–16:00	Ref.	-		Ref.	-	
16:00–24:00	0.932	(0.626, 1.387)	0.728	0.735	(0.433, 1.246)	0.253
00:00–08:00	0.835	(0.527, 1.323)	0.443	0.661	(0.345, 1.268)	0.213
Bystander-witnessed	Ref.	-	<0.001	Ref.	-	0.019
Bystander unwitnessed	0.377	(0.257, 0.552)		0.545	(0.329, 0.906)	
Bystander CPR (+)	Ref.	-	0.168	Ref.	-	0.938
Bystander CPR (−)	0.762	(0.517, 1.121)		0.980	(0.581, 1.651)	
Bystander defibrillation (+)	Ref.	-	0.055	Ref.	-	0.913
Bystander defibrillation (−)	0.526	(0.273, 1.014)		1.060	(0.372, 3.021)	
EMS variables						
Initial cardiac rhythm						
shockable	Ref.	-	<0.001	Ref.	-	<0.001
nonshockable	0.134	(0.091, 0.198)		0.283	(0.160, 0.500)	
Advanced airway						
I-gel/supraglottic	Ref.	-		Ref.	-	
tracheal intubation	1.807	(0.700, 4.670)	0.222	1.403	(0.406, 4.850)	0.593
no advanced airway	2.842	(1.881, 4.294)	<0.001	1.877	(1.067, 3.301)	0.029
Mechanical chest compression (+)	Ref.	-	<0.001	Ref.	-	<0.001
Mechanical chest compression (−)	4.335	(2.616, 7.184)		3.772	(2.022, 7.037)	
Epinephrine administration (+)	Ref.	-	0.209	Ref.	-	0.845
Epinephrine administration (−)	1.387	(0.832, 2.311)		0.932	(0.459, 1.891)	
EMS process time						
EMS response time						
≤8 min	Ref.	-	0.129	Ref.	-	0.253
>8 min	0.748	(0.515, 1.088)		0.734	(0.432, 1.247)	
EMS scene time						
≤15 min	Ref.	-	0.017	Ref.	-	0.222
>15 min	0.627	(0.427, 0.921)		0.715	(0.417, 1.226)	
EMS transport time						
≤8 min	Ref.	-	<0.001	Ref.	-	<0.001
>8 min	2.316	(1.629, 3.294)		3.202	(1.981, 5.175)	

Values are presented as the odds ratio (OR) and 95% confidence interval (95% CI).

**Table 4:** Factors associated with survival to discharge after OHCA in the urban group.

Variable	Univariate Analysis			Multivariate Analysis		
	OR	95% CI	*p* Value	OR	95% CI	*p* Value
Patient variables						
Age ≤ 65 years	Ref.	-	<0.001	Ref.	-	<0.001
Age > 65 years	0.280	(0.188, 0.417)		0.322	(0.187, 0.554)	
Sex (male)	1.802	(1.151, 2.821)	0.010	1.386	(0.761, 2.523)	0.286
Sex (female)	Ref.	-		Ref.	-	
Medical history						
cardiac disease (+)	Ref.	-	0.006	Ref.	-	0.003
cardiac disease (−)	0.526	(0.333, 0.831)		0.388	(0.209, 0.720)	
Bystander variables						
Arrest location						
residence (home)	0.295	(0.194, 0.447)	<0.001	0.460	(0.261, 0.811)	0.007
public	Ref.	-		Ref.	-	
others	0.502	(0.257, 0.983)	0.044	0.193	(0.048, 0.770)	0.020
EMS call time (24 h)						
08:00–16:00	Ref.	-		Ref.	-	
16:00–24:00	0.893	(0.575, 1.388)	0.616	0.704	(0.390, 1.270)	0.244
00:00–08:00	0.898	(0.543, 1.486)	0.676	0.746	(0.367, 1.519)	0.420
Bystander-witnessed	Ref.	-	<0.001	Ref.	-	0.062
Bystander unwitnessed	0.414	(0.274, 0.624)		0.590	(0.339, 1.027)	
Bystander CPR (+)	Ref.	-	0.413	Ref.	-	0.804
Bystander CPR (−)	0.838	(0.549, 1.280)		1.075	(0.607, 1.904)	
Bystander defibrillation (+)	Ref.	-	0.120	Ref.	-	0.814
Bystander defibrillation (−)	0.588	(0.302, 1.147)		1.138	(0.388, 3.333)	
EMS variables						
Initial cardiac rhythm						
shockable	Ref.	-	<0.001	Ref.	-	0.033
nonshockable	0.149	(0.094, 0.235)		0.477	(0.241, 0.942)	
Advanced airway						
I-gel/supraglottic	Ref.	-		Ref.	-	
tracheal intubation	2.018	(0.759, 5.366)	0.159	1.556	(0.448, 5.402)	0.487
no advanced airway	2.475	(1.564, 3.918)	<0.001	2.065	(1.138, 3.747)	0.017
Mechanical chest compression (+)	Ref.	-	<0.001	Ref.	-	<0.001
Mechanical chest compression (−)	4.764	(2.734, 8.303)		3.932	(2.015, 7.674)	
Epinephrine administration (+)	Ref.	-	0.497	Ref.	-	0.655
Epinephrine administration (−)	1.210	(0.698, 2.098)		0.840	(0.391, 1.805)	
EMS process time						
EMS response time						
≤8 min	Ref.	-	0.199	Ref.	-	0.171
>8 min	0.752	(0.487, 1.161)		0.657	(0.360, 1.200)	
EMS scene time						
≤15 min	Ref.	-	0.140	Ref.	-	0.711
>15 min	0.734	(0.487, 1.107)		0.897	(0.505, 1.593)	
EMS transport time						
≤8 min	Ref.	-	<0.001	Ref.	-	<0.001
>8 min	2.896	(1.960, 4.279)		3.521	(2.075, 5.975)	

Values are presented as the odds ratio (OR) and 95% confidence interval (95% CI).

**Table 5:** Factors associated with survival to discharge after OHCA in the rural group.

Variable	Univariate Analysis			Multivariate Analysis		
	OR	95% CI	*p* Value	OR	95% CI	*p* Value
Patient variables						
Age ≤ 65 years	Ref.	-	<0.001	Ref.	-	0.015
Age > 65 years	0.064	(0.019, 0.220)		0.116	(0.020, 0.659)	
Sex (male)	3.362	(1.125, 10.050)	0.030	1.040	(0.175, 6.188)	0.966
Sex (female)	Ref.	-		Ref.	-	
Medical history						
cardiac disease (+)	Ref.	-	0.692	Ref.	-	0.128
cardiac disease (−)	1.284	(0.373, 4.415)		7.355	(0.563, 96.033)	
Bystander variables						
Arrest location						
residence (home)	0.144	(0.055, 0.373)	<0.001	1.088	(0.197, 6.028)	0.923
public	Ref.	-		Ref.	-	
others	0.354	(0.097, 1.296)	0.117	0.000	(0.000, -)	0.998
EMS call time (24 h)						
08:00–16:00	Ref.	-		Ref.	-	
16:00–24:00	1.072	(0.418, 2.749)	0.885	1.467	(0.319, 6.754)	0.623
00:00–08:00	0.636	(0.193, 2.100)	0.458	0.571	(0.080, 4.095)	0.577
Bystander-witnessed	Ref.	-	0.003	Ref.	-	0.123
Bystander unwitnessed	0.149	(0.043, 0.516)		0.262	(0.048, 1.438)	
Bystander CPR (+)	Ref.	-	0.271	Ref.	-	0.577
Bystander CPR (−)	0.576	(0.216, 1.538)		0.655	(0.148, 2.895)	
Bystander defibrillation (+)	Ref.	-	0.999	Ref.	-	0.999
Bystander defibrillation (−) ^1^	54,229,720.413 ^1^	(0.000, -)		101,892.415	(0.000, -)	
EMS variables						
Initial cardiac rhythm						
shockable	Ref.	-	<0.001	Ref.	-	<0.001
nonshockable	0.044	(0.017, 0.116)		0.028	(0.005, 0.159)	
Advanced airway						
I-gel/supraglottic	Ref.	-		Ref.	-	
tracheal intubation	0.000	(0.000, -)	0.999	0.000	(0.000, -)	0.999
no advanced airway	3.162	(1.195, 8.364)	0.020	0.580	(0.057, 5.910)	0.646
Mechanical chest compression (+)	Ref.	-	0.043	Ref.	-	0.252
Mechanical chest compression (−)	3.552	(1.040, 12.133)		3.220	(0.435, 23.822)	
Epinephrine administration (+)	Ref.	-	0.241	Ref.	-	0.236
Epinephrine administration (−)	2.406	(0.555, 10.431)		4.174	(0.392, 44.425)	
EMS process time						
EMS response time						
≤8 min	Ref.	-	0.392	Ref.	-	0.526
>8 min	1.458	(0.615, 3.461)		1.647	(0.352, 7.707)	
EMS scene time						
≤15 min	Ref.	-	0.014	Ref.	-	0.039
>15 min	0.160	(0.037, 0.691)		0.076	(0.006, 0.883)	
EMS transport time						
≤8 min	Ref.	-	0.103	Ref.	-	0.045
>8 min	2.085	(0.863, 5.041)		4.741	(1.035, 21.706)	

Values are presented as the odds ratio (OR) and 95% confidence interval (95% CI). ^1^ ORs should be interpreted carefully since no patient survived to discharge in the bystander defibrillation (+) group.

## Data Availability

Raw data were generated by national fire agencies in Korea. The use of data requires contacting and obtaining permission from the national fire agencies. Derived data supporting the findings of this study are available from the corresponding author (SHK) upon reasonable request.

## Appendix A

1. Demographics and characteristics of the 1:1 matched study population
2. Survival outcomes of the 1:1 matched study population
3. Factors associated with survival to discharge after OHCA in the Busan vs. rural groups
4. Factors associated with survival to discharge after OHCA in the Ulsan vs. rural groups
5. Factors associated with survival to discharge after OHCA in the Changwon vs. rural groups
